# Impacts of Different Tree Species of Different Sizes on Spatial Distribution of Herbaceous Plants in the Nigerian Guinea Savannah Ecological Zone

**DOI:** 10.1155/2015/106930

**Published:** 2015-11-05

**Authors:** Toma Buba

**Affiliations:** Department of Biological Sciences, Faculty of Science, Abubakar Tafawa Balewa University, PMB 0248, Bauchi, Nigeria

## Abstract

This study was aimed at finding the impacts of different tree species and individual trees of different sizes on species richness, diversity, and composition of the herbaceous layer. All the three tree species have greatly increased species richness and diversity both within and outside their crown zones compared with the open grassland. Both species richness and diversity were found to be higher under all the three tree species than outside their crowns, which was in turn higher than the open field. *Daniella oliveri* has the highest species richness and diversity both within and outside its crown zone followed by *Vitellaria paradoxa* and then *Parkia biglobosa*. The result also revealed that the same tree species with different sizes leads to different herbaceous species richness, diversity, and composition under and around the trees' crowns. *P. biglobosa* and *V. paradoxa* trees with smaller sizes showed higher species richness and diversity under their crowns than the bigger ones. The dissimilarity of species composition differs between the inside and outside crown zones of the individuals of the same tree species and among the different trees species and the open field.

## 1. Introduction

Vegetation with its biodiversity is a crucial part of the Earth Systems (soil, water, and atmosphere), offering essential services to both the ecosystem and human societies [1]. Vegetation supports the functions of soil, which include decomposition, nutrient cycling, soil respiration, invasion resistance, and ecosystem stability. Soil biodiversity in turn provides many ecosystem services essential to mankind and the environment, such as the support of primary production, control of pests and diseases for man and his animals and crops, and the avoidance of environmental contamination through cycling of dead biomass. Also, soil biota plays a significant role in determining the soil physicochemical properties. Plants, in particular, play an important role in shaping soil profile with the diverse architecture of their root systems, being the center of soil-plant-microbial interactions [2].

Vegetation also contributes significantly to the control of soil erosion, which is a serious threat to ecosystem functioning in many parts of the world. It was reported that the magnitude of impact of plants in rendering ecosystem services, particularly in controlling soil erosion, depends on structural architecture of the plant's morphologies (roots systems, crown shape and size, etc.), which in turn depends on the species [3–6]. Hence, diversity within vegetation has been shown to have remarkable effects on ecosystem functions and stability. More often, increasing biodiversity enhanced ecosystem productivity and stability. For example, Seitz et al. [6] report that tree monocultures in afforestation have only limited mitigation potential for soil losses and that there is growing evidence that higher species richness can reduce soil erosion.

Vegetation is also of crucial benefits to the human societies. Many authors report the effectiveness of vegetation in protecting man-made structures like roads, embankments, man-made dams, and pastures that support a significant part of food production, and so forth [1, 7]. It is then of great importance to understand the consequences of the worldwide losses of plant species diversity for the ecosystem services in many parts of the world. Of fundamental importance amongst these are issues of biodiversity, biofuels/energy security, climate change, food security, human enterprises, land degradation, and water security [1, 2, 6].

The importance of vegetation in controlling soil erosion, protecting water resources, and supporting carbon cycle has been widely reported. In controlling soil erosion, vegetation is described as the key factor in the processes. Hence, many agroenvironments get protection against top soil loss from surrounding vegetation and intercropping, safe-guarding food production, and security worldwide [8].

Vegetation also plays an important role in water and sediment dynamics of rivers downstream. It has a significant impact on the water and sediment transport in headwater catchments because the vegetation attributes can cause obstructions which generate drag on the flowing water. This drag slows water discharge and reduces the stream flow velocity which in turn influences the sediment dynamics of the flow. This hydrological response happens also as a result of the vegetation ability to enhance infiltration thereby reducing crusting in the soil surface [9–11]. Hence, vegetation was found to protect water bodies like dams, streams, and rivers from sediment for the benefits of human societies in particular and the ecosystem in general [12–14].

Vegetation also contributes immensely to stocking of soil organic carbon, which is essential for good soil structure and nutrient availability that support the soil biota and aboveground productivity. Higher organic carbon concentration and storage were reported under vegetated lands compared with those in bare fields [15, 16].

The vegetation in the drier tropical savannas is patchy in distribution with high plant cover (shrub patches) interspersed in a low-cover herbaceous matrix (intershrub areas). This is the result of spatial heterogeneity in local topography and soil microtopography, physicochemical properties, and water distribution [17]. The result is that there are wide ranges of variabilities of the ecosystem services from different local or microenvironment. For example, Cerdà [18] reported that vegetated patches have positively affected surface runoff, erosion, infiltration rates, and deep wetting fronts compared with bare patches. For this reason, different plant communities differ in their resistance to land degradation, and land degradation is thus highly variable among areas [19].

Trees create a unique microenvironment around them [20] by positively modifying their underneath soil physicochemical properties and by direct influence on sunlight and atmospheric conditions [21]. Transpiration and canopy are the main factors affecting air temperature, wind speed, the quantity and quality of light, and soil temperature, pH, moisture, and nutrient availability [22]. The change in soil physicochemical properties depends on the litter quality and quantity and the canopy architecture, which in turn depends on the tree species [23]. As different species of the herbaceous layer respond differently to soil nutrient status and other environmental constraints, the trees are then seen as one of the main factors that influence understory floristic species richness, diversity, and composition. These lead to the notion of zone of influence or ecological fields created by individual trees, especially those with isolated canopy in the savannah [22, 24].

The tree crown interacts with solar radiation through absorption and scattering. These processes vary with the leaf structure, size, shape, orientation, distribution, and age and density of the leaf layers, as well as crown volume [25]. All these critical parameters are different among different tree species and individuals of the same species with different age or size [25–28]. Light availability generally affects plant performance but the degree and pattern of these effects may be species specific [22, 29]. Limiting light availability also helps maintain soil moisture [30]. In this regard, trees have strong filtering ability, which determines the patterns floristic composition of the herbaceous layer [31, 32].

There are also considerable differences in the pattern of rainfall reaching the ground in many plant communities, because of the interception by the tree crown. The water may be subsequently transferred to the soil by channeling down the main stem or by dripping from the branches. The stem flow is greatly enhanced by branches and leaves, which are inclined upward. Stem flow produces a great concentration of water around the base of the trunk, which may be significant ecologically. These morphological features may be powerful ecological factor in plant distribution [25]. Interception of rainfall by trees and the subsequent stem flow induces pattern of soil wetting nearby, for smaller plants [33]. An enhanced concentration of water and nutrients creates favorable microenvironments, which in turn enhances the establishment, growth, and persistence of the herbs beneath [20, 21].

Trees also tend to affect properties of soil around them through litter-fall input, which are relative to the tree species and individual sizes [21, 34, 35]. Studies revealed that soils were sandier and slightly acidic under canopies of medium and large trees compared to small trees, which have slightly alkaline soils. Soils in the tree interspaces have significantly higher silt and clay content than beneath trees [36]. Thus, distribution of general soil fertility, organic matter, nitrogen, phosphorus, and potassium, as well as microbial activities, becomes spatially and vertically concentrated under the tree canopy [20]. Elsewhere, soils under canopies were found to have significantly higher levels of organic matter, calcium, magnesium, and pH than those in open grassland [34].

The general patterns of response of herbaceous layer under tree canopy to increase in nutrient availability, especially nitrogen, often include initial increase in cover layer as a whole, decrease in species richness from loss of relatively numerous nitrogen-efficient species, and decrease in species evenness from increasing dominance of few high nitrogen-requiring species [29, 37]. However, the effects of trees on the associated understory herbaceous productivity vary with the environment or the climatic conditions [38]. In addition, different herbaceous plant species will respond differently to different types of tree canopies; therefore, results of study from one area with specific tree species cannot be extrapolated to other areas with different trees and herbaceous plant species composition.

Although numerous studies have demonstrated that the species composition and diversity of understory flora can be influenced by tree canopy [39], such a study is almost virtually absent in the Nigerian dry lands. This study was carried out to find the impacts of different tree species and individuals of different sizes on composition, richness, and diversity of the herbaceous layer. The findings of such studies are indispensable tool in conservation and management of practices of grasslands. The information on the pattern of impact of trees on their environment will be beneficial in restoring diversity in the dry lands, especially with regard to choosing tree species for reforestation or afforestation programmes.

The general aim of this study was to find how tree species affect the composition of the herbaceous layer underneath; specifically, to find whether different tree species affect the underneath herbaceous composition in different ways; and also to find whether individuals of the same trees species but of different sizes affect the herbaceous layer in different way in terms of species composition, richness, and diversity.

## 2. Methodology

### 2.1. Study Area

The study area was the Yelwa campus of Abubakar Tafawa Balewa University, Bauchi, located at latitude 100 17I North, longitude 80 49I East, at the altitude of 690.2 M above sea level in the northern guinea savanna ecological zone of Nigeria [40]. The soils here are generally classified as Alfisols [41]. The soil is highly weathered and fragile with low activity clays, thus making their fertility decline under continuous arable cropping. The excessive soil nutrient mining and degradation have been reported to be one of the most biogeophysical constraints in this region [42]. The climate is characterized by rainy season that starts in April and ends in October, with the amount of rainfall of 1300 mm per annum [43], with the lowest mean monthly relative humidity at about 29% [44]. The month of April is the hottest month of the year with mean minimum and maximum temperature of 13.7°C and 30.11°C, respectively [45]. The vegetation type is open woodland, dominated with tall grasses ranges between one and three meters (m) high in open areas and trees (up to 15 m high) usually with short boles, broad leaves, and isolated crowns. This vegetation is subjected to fierce wild fires almost annually in the dry season. It is therefore predominated by fire-resistant species [44]. Species such as* Isoberlinia doka* and* I. tomentosa* form the bulk of the scattered woodland in the northern guinea savannah. Also found are locust bean tree (*Parkia biglobosa*) and shea butter tree (*Vitellaria paradoxa*) [46].

### 2.2. Floristic Data Collection

**Figure 1:**
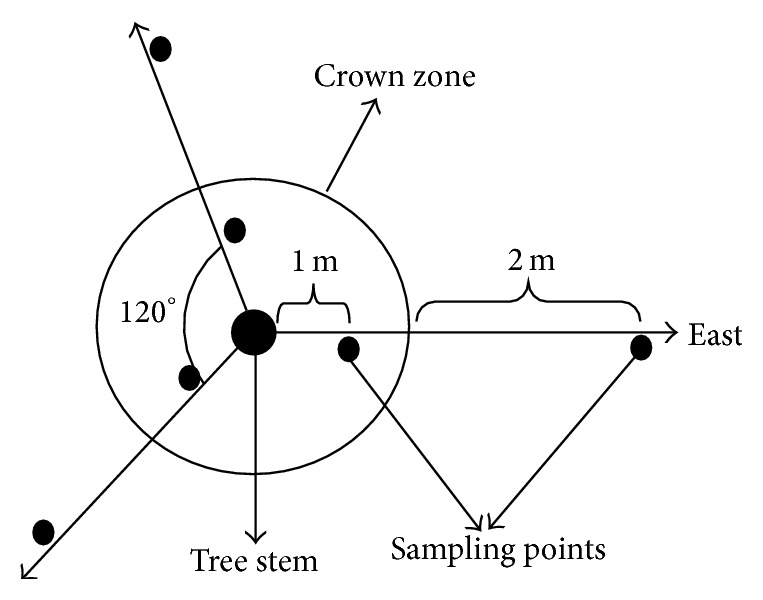
Diagram of the sampling design.

The tree species used in this study were* Parkia biglobosa* (Jacq.) Benth.,* Daniella oliveri* (Rolfe) Hutch. & Dalziel, and* Vitellaria paradoxa* C.F. Gaertn. To collect the floristic data, the circumference of each tree crown zone was roughly divided into three sections, that is, 120° sectors. The dividing lines of the sectors were extended to the distance of 3 meters outside the crown zone from the base of the main stem (Figure 1). A quadrat of size 50 by 50 cm was laid at a standard distance of one meter from the main stem along each of the three dividing lines of the crown zone. The sampling was repeated at the distance of two meters away from the crown zone, but still on the lines of divisions. Thus, there were three samples within the crown zone and three outside the crown zone of each tree. The sampling was repeated for five individual trees of the same species, that is, fifteen samples each for within and outside crown zones of each tree species. Fifteen samples were also taken from the open field, which was a distance of at least more than 10 meters away from the nearest tree. The number of individuals of each plants species within the quadrat was recorded. In addition, specimens of each species were collected and subsequently identified in the Abubakar Tafawa Balewa University herbarium.

To compare the impact of tree size on the understory herbaceous layer, some individuals of* V. paradoxa* and* P. biglobosa* were subjectively categorized based on their sizes as big and small for the purpose of this study, but* D. oliveri* were not categorized on sizes because they were found to be almost of the same size (Table 1). To estimate their sizes, the averages of the shortest and the longest axis of the tree crown diameter were measured in meters. Tree crown diameter is known to be directly proportional to the overall size of the tree and therefore good estimate of the tree size [47, 48].

### 2.3. Data Analyses

All plant species parameters were calculated using the software Community Ecology Parameter Calculator (ComEcoPaC) Version 1.0 [49]. According to the designer, formulae used by this software were as follows.

*Shannon-Wiener Diversity Index (H*′). The Shannon-Wiener Diversity Index (*H*′) is as follows:

(1) $$\begin{array}{c} H^{\prime }=\overset{S}{\underset{i-1}{\sum }}p_{i}\cdot \log_{2}p_{i}, \end{array}$$

where *S* is the species richness (number of species), *p*_*i*_ is the proportion of species *i*, and *p*_*i*_ = *n*_*i*_/*N* (*n*_*i*_ is the abundance of species *i*; *N* is the total abundance). 

*Evenness (E*). Evenness is represented as follows:

(2) $$\begin{array}{c} E=\frac{H^{\prime }}{H_{max}}, \end{array}$$

where *H*_max_′ = log_2_⁡*S*  and *H*_min_′ = −((*N* − *S* + 1)/*N*)log_2_⁡((*N* − *S* + 1)/*N*) + ((*S* − 1)/*N*)log_2_⁡*N*.

*Jaccard's Similarity Index*. Jaccard's similarity index is represented as follows:

(3) $$\begin{array}{c} Ja=\frac{S_{12}}{S_{1}+S_{2}-S_{12}}, \end{array}$$

where *S*_12_ is the number of species present in both samples (joint occurrences) and *S*_1_ (*S*_2_) is the number of species present in sample one (sample two).

 Jaccard's dissimilarity index = 1 − similarity index [50].

*One-Sample t-Test* was carried out toestimate* standard deviation* and* Standard Error of the Mean* for all the species parameters using Minitab version 17.2.1 (©2013, 2015 Minitab., Inc.).

## 3. Result

The result revealed that all the three tree species have greatly increased species richness both within and outside their crown zones, which ranged from 31 to 39 compared with the open field, which was just 20 (Table 2).* D. oliveri* has the highest species richness both within and outside its crown zone followed by* V. paradoxa* and then* P. biglobosa*. There were a slightly higher number of species inside than outside crown zones of all the trees. Species diversity also was found to be higher inside than outside crown zones of all the trees and the open field. Species diversity was highest under* D. oliveri*. Species evenness seems to be unaffected by the trees.

The dissimilarity of species composition differs between the inside and outside crown zones of the individuals of the same tree species and among the different trees species and the open field (Table 3). Higher dissimilarity was found between the inside and outside crown zone of* P. biglobosa* (0.55). The least dissimilarity was between the inside and outside crown zone of* D. oliveri* (0.29), while for that of* V. paradoxa* the value was 0.5. Comparisons between the open field and the inside and outside crown zones of all the tree species also revealed great differences in the species composition. The values range from 0.47 to 0.63. However, higher dissimilarities were found between the open field and the inside crown zones than the outside of crown zones of all the tree species.

The study also showed that species richness was slightly higher in the inside and outside crown zones of individual trees with small size compared with the big trees of* P. biglobosa* and* V. paradoxa* (Table 4). Higher species diversity was also found to be associated with both inside and outside crown zone of small* P. biglobosa* compared with bigger ones while, for inside crown zone of* V. paradoxa*, higher diversity was found with big trees than small trees. However, there was higher diversity outside crown zone of small* V. paradoxa* compared to the bigger ones.

The similarity or dissimilarity of understory herbaceous species composition also differs with different tree sizes (Table 5). The dissimilarity value between the inside crown zones of big and small* V. paradoxa* was 0.5, while the outside crown zones have dissimilarity of 0.66. In* P. biglobosa*, dissimilarity value between the inside crown zones of big and small individual trees was 0.62, while the outside crown zones of big and small trees have dissimilarity value of 0.33.

## 4. Discussion

It is already well documented that trees affect their environment through shading, rainfall interception, interference with light penetration, and the quantity and quality of litter they produce that change soil physical and chemical properties. The amount of these impacts through interference with sunlight availability and soil nutrient amplification also depends on the tree species and size of the individual [51]. This brings about improved soils fertility and structure below their crowns; improved water relations of plants in their shades; and increased competition for light, soil moisture, and nutrients in the herb layer, which generates spatial environmental heterogeneity at different scales. The spatial variability of microenvironments created by the trees leads to differences in floristic parameters of the herbaceous layer [52], because plant species respond individualistically to environmental variables and, therefore, to tree influences [53, 54]. The nature and intensity of the effect of these trees may also depend on their leaf area, canopy architecture, patterns rooting system, and so forth, which in turn depend on the tree species in question [51, 53].

In this study, all the trees were also found to affect the distribution and composition of herbaceous layer under and around their crowns in comparison with the open field. There were increased species richness and diversity under and outside the trees canopy, which was greater than that of the open field. In contrast, some studies suggested that the response of herbaceous layer under tree canopy led to decrease in species richness and diversity because increase in soil nitrogen input will lead to the loss of many nitrogen-efficient species which brings about dominance of few high nitrogen-requiring species [29, 37]. Studies in a forest by Vockenhuber et al. [55] also showed a negative response of species richness to increasing canopy cover.

This study also revealed that the impacts of the trees on herbaceous species richness and diversity are different under different tree species. In the increasing order of impact, the trees are* Parkia biglobosa*,* Vitellaria paradoxa*, and* Daniella oliveri*. This order was also observed to be relative to the size of their crowns (cover) in decreasing order. This means that the greater the crown size or cover, the lesser the species richness and diversity under them. Here, the implication in terms of light availability is that the smaller crowns will allow more sunlight to reach the ground in the midday and allow full sunlight under them in the morning and evening. Many studies also revealed increased species richness of the herb layer with increasing tree diversity. This is because herbaceous layer responds differently to different microenvironment created by different tree species, that is, by increasing environmental heterogeneity or by creating environmental conditions that are favourable to a greater number of the herbaceous species [52, 55].

It is also interesting to note that species richness and diversity were found to be higher under all the three tree species than outside their crowns. However, reduction of light intensity by tree crown was known to suppress productivity in many agricultural experiments. The influence of trees on species richness and diversity is considered to be associated with two most important factors: the altered soil conditions and light levels [56]. It is also important to note that, in almost all studies on the impact of trees on herb layer, no quantitative measurements are taken on the tree crowns and the light they intercept was not quantified. Hence, interpretation of the response of the herbaceous layer remains largely subjective.

The tree size was also known to be a factor that determines degree of the tree impact on the trees' environments [53, 54]. In the savanna, where trees grow with isolated crowns, trees with smaller crown size will be expected to receive more or full sunlight under their canopy than trees with bigger crown diameter, especially in the morning and evening. In this study,* P. biglobosa* and* V. paradoxa* trees with smaller sizes showed higher species richness and diversity under their crowns than the bigger ones. Again, small trees are expected to have less nutrient availability and greater sunlight penetration under their crowns [54]. These suggest that abundance of sunlight may reduce or suppress dominance by few high nutrient-requiring species because of increase in competitive ability of the species that are sun lover with low nutrient demand. The combine effects of optimal nutrient availability and sunlight may be the factors that bring about increase of species richness and diversity. Some studies also reported that smaller trees have a different effect on the understory vegetation than large trees or open grassland [57].

Different plant species have different optimal environmental requirement and also different local environmental conditions were created by different tree species or the same tree species but with different sizes. Each of these heterogeneous microhabitats that are specific for each tree species and size will be optimal or near optimal for some species of the grass layer but not others. The response of the herbaceous layer to sunlight and nutrient availability is also species specific. One species will find the environment more suitable than others will; hence, the most favored species will outperform the less favored ones. Therefore, different microenvironment created by different tree species or by individuals of different sizes will be occupied by combination of different species with different degree of abundance. It is therefore expected that herbaceous species composition will differ with respect to tree species and size and the open grassland, which is in agreement with Ludwig et al. [54]. It has also been suggested by Vockenhuber et al. [55] that the environmental heterogeneity created by higher tree diversity may lead to higher small-scale heterogeneity of site conditions and consequently to higher herbaceous species diversity.

The three tree species used in this study create herbaceous plant communities with different species composition underneath and around their crown zones. Also, the same tree species of different sizes of individual also created different herbaceous species composition beneath their crown. This means that the ground herbaceous layer also responded in a different way in combination and relative abundance with respect to the trees' microenvironment created by different tree species leading to high dissimilarity values among the three tree species. Elsewhere, studies showed that species composition of the herbaceous layer might change along gradients extending from the bole to the canopy and the open grassland [53, 56].

Finally, this study revealed that different tree species with different sizes lead to different herbaceous species richness, diversity, and composition under and around their crowns. However, generally, studies on tree-grass interactions in savannas should include consideration of elements of competition and facilitation by the trees, climate and seasonal variability, stages of succession, soil type, tree density, and varying other biotic and abiotic complexities in both time and space[52, 53]. Therefore, the result of this study is not conclusive.

## 5. Conclusion

All the three tree species have greatly increased species richness and diversity both within and outside their crown zones compared with the open grassland. Both species richness and diversity were found to be higher under all the three tree species than outside their crowns, which was higher than the open field.* D. oliveri* has the highest species richness and diversity both within and outside its crown zone followed by* V. paradoxa* and then* P. biglobosa*. The result also revealed that the same tree species with different sizes leads to different herbaceous species richness, diversity, and composition under and around the trees' crowns.* P. biglobosa* and* V. paradoxa* trees with smaller sizes showed higher species richness and diversity under their crowns than the bigger ones. The dissimilarity of species composition also differs between the inside and outside crown zones of the individuals of the same tree species and among the different tree species and the open field.

## Figures and Tables

**Table 1:** The sizes of the individual trees that were used in the study for comparison.

Tree species	Big tree	Small tree
	Crown diameter	Crown diameter
*V. paradoxa*	16.6 m	8.8 m
	14.5 m	8.3 m
	12.4 m	7.7 m

*P. biglobosa*	20 m	13.6 m
	24.5 m	12.4 m
	20.1 m	12.8 m

**Table 2:** Species richness, diversity, and evenness from inside and outside crown zones and the open field, with the estimates of their standard deviation (StDev) and Standard Error of the Mean (SE Mean). PB:* P. biglobosa*; DO:* D. oliveri*; VP:* V. paradoxa*.

	PB inside	PB outside	DO inside	DO outside	VP inside	VP outside	Open field	SE Mean	StDev
Species richness	31	33	39	38	31	29	20	2.39	6.32
Shannon Index	3.92	3.34	4.16	3.73	3.69	3.38	3.39	0.117	0.31
Evenness	0.79	0.66	0.79	0.71	0.74	0.7	0.78	0.0192	0.0508

**Table 3:** Half matrix of dissimilarity of species composition among inside and outside crown zones of the trees and the open field. PB:* P. biglobosa*; DO:* D. oliveri*; VP:* V. paradoxa*.

Jaccard's similarity index	PB outside	DO inside	DO outside	VP inside	VP outside	Open field
PB inside	0.55	0.48	0.53	0.56	0.5	0.62
PB outside		0.36	0.42	0.55	0.32	0.57
DO inside			0.29	0.48	0.49	0.63
DO outside				0.47	0.4	0.51
VP inside					0.5	0.54
VP outside						0.47

**Table 4:** Species richness, diversity, and evenness from inside and outside crown zones of trees with different sizes and the open field, with their standard deviation (StDev) and Standard Error of the Mean (SE Mean). PB: *P. biglobosa*; VP: *V. paradoxa*.

	Small VP inside	Small VP outside	Big VP inside	Big VP outside	Small PB inside	Small PB outside	Big PB inside	Big PB outside	SE Mean	StDev
Species richness	26	24	25	22	23	28	21	27	0.866	2.449
Shannon Index	3.43	3.15	3.77	3.12	3.72	3.26	2.47	2.98	0.148	0.419
Evenness	0.73	0.69	0.81	0.70	0.82	0.68	0.56	0.63	0.0306	0.0865

**Table 5:** Half matrix of similarity and dissimilarity (in parenthesis) of species composition among inside and outside crown zones of the tree of different sizes. PB: *P. biglobosa*; DO: *D. oliveri*; VP: *V. paradoxa*.

Jaccard's similarity index	Small VP outside	Big VP inside	Big VP outside	Small PB inside	Small PB outside	Big PB inside	Big PB outside
Small VP inside	0.43 (0.57)	0.5 (0.5)	0.33 (0.77)	0.4 (0.6)	0.38 (0.62)	0.47 (0.53)	0.43 (0.67)
Small VP outside		0.53 (0.47)	0.44 (0.66)	0.52 (0.48)	0.49 (0.51)	0.36 (0.64)	0.46 (0.54)
Big VP inside			0.57 (0.43)	0.45 (0.55)	0.56 (0.44)	0.44 (0.66)	0.53 (0.47)
Big VP outside				0.41 (0.59)	0.56 (0.44)	0.39 (0.61)	0.58 (0.42)
Small PB inside					0.42 (0.58)	0.38 (0.62)	0.47 (0.53)
Small PB outside						0.32 (0.68)	0.67 (0.33)
Big PB inside							0.37 (0.63)
